# Functional production of human antibody by the filamentous fungus *Aspergillus oryzae*

**DOI:** 10.1186/s40694-020-00098-w

**Published:** 2020-05-28

**Authors:** Hung Hiep Huynh, Naoki Morita, Toshihiro Sakamoto, Takuya Katayama, Takuya Miyakawa, Masaru Tanokura, Yasunori Chiba, Reiko Shinkura, Jun-ichi Maruyama

**Affiliations:** 1 Department of Biotechnology, The University of Tokyo, Tokyo, Japan; 2 Laboratory of Immunology and Infection Control, Institute for Quantitative Biosciences, The University of Tokyo, Tokyo, Japan; 3 Collaborative Research Institute for Innovative Microbiology, The University of Tokyo, Tokyo, Japan; 4 Department of Applied Biological Chemistry, The University of Tokyo, Tokyo, Japan; 5 Biotechnology Research Institute for Drug Discovery, National Institute of Advanced Industrial Science and Technology (AIST), Tsukuba, Ibaraki Japan; 6 Core Research for Evolutional Science and Technology, Japan Agency for Medical Research and Development, Tokyo, Japan

**Keywords:** *Aspergillus oryzae*, Antibody, Neutralization, Fc receptor, *N*-linked glycan

## Abstract

**Background:**

Monoclonal antibodies (mAbs) as biopharmaceuticals take a pivotal role in the current therapeutic applications. Generally mammalian cell lines, such as those derived from Chinese hamster ovaries (CHO), are used to produce the recombinant antibody. However, there are still concerns about the high cost and the risk of pathogenic contamination when using mammalian cells. *Aspergillus oryzae*, a filamentous fungus recognized as a GRAS (Generally Regarded As Safe) organism, has an ability to secrete a large amount of proteins into the culture supernatant, and thus the fungus has been used as one of the cost-effective microbial hosts for heterologous protein production. Pursuing this strategy the human anti-TNFα antibody adalimumab, one of the world’s best-selling antibodies for the treatment of immune-mediated inflammatory diseases including rheumatoid arthritis, was chosen to produce the full length of mAbs by *A. oryzae*. Generally, *N*-glycosylation of the antibody affects immune effector functions such as antibody-dependent cell-mediated cytotoxicity (ADCC) via binding to the Fc receptor (FcγR) on immune cells. The CRISPR/Cas9 system was used to first delete the *Aooch1* gene encoding a key enzyme for the hyper-mannosylation process in fungi to investigate the binding ability of antibody with FcγRIIIa.

**Results:**

Adalimumab was expressed in *A. oryzae* by the fusion protein system with α-amylase AmyB. The full-length adalimumab consisting of two heavy and two light chains was successfully produced in the culture supernatants. Among the producing strains, the highest amount of antibody was obtained from the ten-protease deletion strain (39.7 mg/L). Two-step purifications by Protein A and size-exclusion chromatography were applied to obtain the high purity sample for further analysis. The antigen-binding and TNFα neutralizing activities of the adalimumab produced by *A. oryzae* were comparable with those of a commercial product Humira^®^. No apparent binding with the FcγRIIIa was detected with the recombinant adalimumab even by altering the *N*-glycan structure using the *Aooch1* deletion strain, which suggests only a little additional activity of immune effector functions.

**Conclusion:**

These results demonstrated an alternative low-cost platform for human antibody production by using *A. oryzae*, possibly offering a reasonable expenditure for patient’s welfare.

## Background

Antibody (or immunoglobulin) is a macro-molecule consisting of four polypeptides. Two pairs of identical heavy chains and light chains form a “Y” shape structure through the disulfide bonds (Additional file 1: Fig. S1). Two tops of the “Y” shape contain the fragment antigen-binding region (Fab) including the variable domain (Fv) of the light and heavy chains. The amino acid sequence of this variable region varies greatly among different antibodies, which gives the antibody its specificity for binding to the antigen. The remaining of “Y” is called the fragment crystallizable region (Fc) and can be bonded to the surface of lymphocytes by the endogenous Fc receptors [1]. Antibodies play a crucial role in the immune system to protect the body from the infection. In the global biopharmaceutical market antibodies, especially immunoglobulin G (IgGs), have held the largest contribution. They are recombinantly produced for therapeutic treatment such as cancer and autoimmune diseases [2]. For manufacturing the antibody, among the mammalian platforms, the Chinese hamster ovary (CHO) cell line is the most widely used [3]. However, there are some concerns about the high cost and the risk of contamination of human pathogens. The high demand and expensiveness of antibodies have encouraged the development of biosimilar molecules produced by different hosts, which possess comparable characteristics to the already approved biopharmaceuticals [4]. Thus, these alternative antibodies are predicted to play a vital role in biomedical market in the coming years and to significantly boost the biological therapy by extending more options for treatment processes [5].

Microbial expression system such as bacteria and fungi have been investigated due to their capacity to produce recombinant proteins with low-cost, simple culturing process and easy genetic modification [6]. *Escherichia coli* is the most popular system for recombinant protein production among bacteria. However, the recombinant protein is typically accumulated in the bacterial cytoplasmic compartment, resulting mostly in inclusion bodies. The recovery step to obtain functional proteins by complete denaturation and refolding is not highly efficient. The lack of ability to generate the disulfide bond in the full-length antibody structure is also a drawback of bacterial platforms. Thus, bacteria are preferred to produce the antibody fragments such as dAb (single domain antibody) and scFv (single chain fragment variable) [6]. Yeasts have a better proficiency to secrete and to process the disulfide bond of recombinant proteins, and thus they have been used for producing various recombinant proteins for food and industrial application [7, 8]. Yeast species such as *Saccharomyces cerevisiae*, *Pichia pastoris* and *Ogataea minuta* were reported for recombinant production not only of antibody fragments [9] but also of full-length antibodies [10–12]. Besides yeasts, filamentous fungi such as *Aspergillus* and *Trichoderma* species are also expected to be favored hosts for the recombinant production of antibodies due to their high abilities to secrete large amounts of proteins. However, the antibody production by filamentous fungi is less well investigated, as full-length antibody production has been reported only once using *Aspergillus niger* var. *awamori* [13] in spite of a number of reports of antibody fragment production [14]. Hence, more efforts need to be paid to unlock the true potential of filamentous fungi.

One of the most important activities of IgG is neutralization, the specific binding to neutralize the antigen, which depends on the structure of Fab region [15]. Another important activity of IgG is the effector functions in antibody-dependent cellular cytotoxicity (ADCC) to recruit the cytotoxic cells such as natural killer cells through the Fc receptor—FcγRIIIa [16]. This feature is taken by the Fc moiety of IgG, which appears in most of therapeutic approved antibodies [17]. In addition, the Fc region helps to protect the IgG from lysosomal degradation by endothelial cells and prolongs the IgG residence time in the vessel circulation to 2–4 weeks, while the antibody fragments only containing the Fab region have the retention time less than 24 h [18]. Consequently, the longer period of antigen binding in the antibody with Fc region helps to improve the efficiency of antibody in each therapeutic treatment [19]. Therefore, the production of the full-length IgG may be more preferred in the production of biopharmaceutical antibody. The full-length antibody IgG contains an *N*-linked glycosylation site in the Fc region at Asn297 position. The *N*-glycan structure contributes to the effector functions in ADCC through Fc receptor on the surface of immune cells [20]. For therapeutic antibody production, it is necessary to genetically modify the host cells for mimicking the mammalian *N*-glycosylation to increase the efficiency and avoid unwanted immunological response [21]. Yeasts and filamentous fungi possess *N*-glycan with the high-mannose structure initiated by the α-1,6-mannosyltranferase (Och1) in the Golgi [22], which differs from the complex-type structure containing sialic acid, galactose, fucose and *N*-acetylglucosamine in human IgG [23]. After the addition of the mannose unit by Och1, *N*-glycan can be elongated by multi-enzymes of Mnn1, Mnn2, Mnn4, Mnn5 and Mnn6 [22]. Therefore, the deletion of the *och1* gene is often the first step in glycoengineering strategies [23–26]. Further modification of glycosylation by heterologously expressing glycosidases, glycosyltranferases and sugar transporters allowed for producing IgGs with the humanized *N*-glycan structure in yeasts [27], while glycoengineering has not yet been performed for IgG production in filamentous fungi.

The filamentous fungus *Aspergillus oryzae* is listed as Generally Regarded As Safe (GRAS) by the U.S. Food and Drug Administration (FDA) due to more than a thousand years of use in Japanese traditional food fermentation [28]. The fungus has been used as a host for heterologous protein production due to the ability to secrete large amounts of proteins into the culture medium [29], and the production of a hetero-oligomeric protein neoculin with the disulfide bond was reported [30]. Thus, *A. oryzae* is expected to be a high potential host for industrial antibody production. Aiming to introduce an adequate platform for industrial antibody production, herein for the first time, we produced adalimumab by *A. oryzae*. Adalimumab is an antibody (IgG) that binds specifically to an inflammatory cytokine, human TNFα, and the antibody has been used in the therapy of the chronic inflammatory diseases including rheumatoid arthritis. Among the biotherapeutic antibodies, Humira^®^, a commercial product of adalimumab, has led the list of top-selling pharmaceutical products since 2012 and achieved $19.9 billion of global sales in 2018 [31]. Moreover, in this study, the initial attempt for modifying the *N*-glycosylation by deleting the *och1* orthologous gene in *A. oryzae* was performed to analyze the effect of the *N*-glycan to Fc receptor FcγRIIIa. These results provided the basis for the development of therapeutic antibodies with cost reduction and easy manufacturing by *A. oryzae*.

## Results

### Adalimumab production in the culture supernatant of *A. oryzae*

**Fig. 1:**
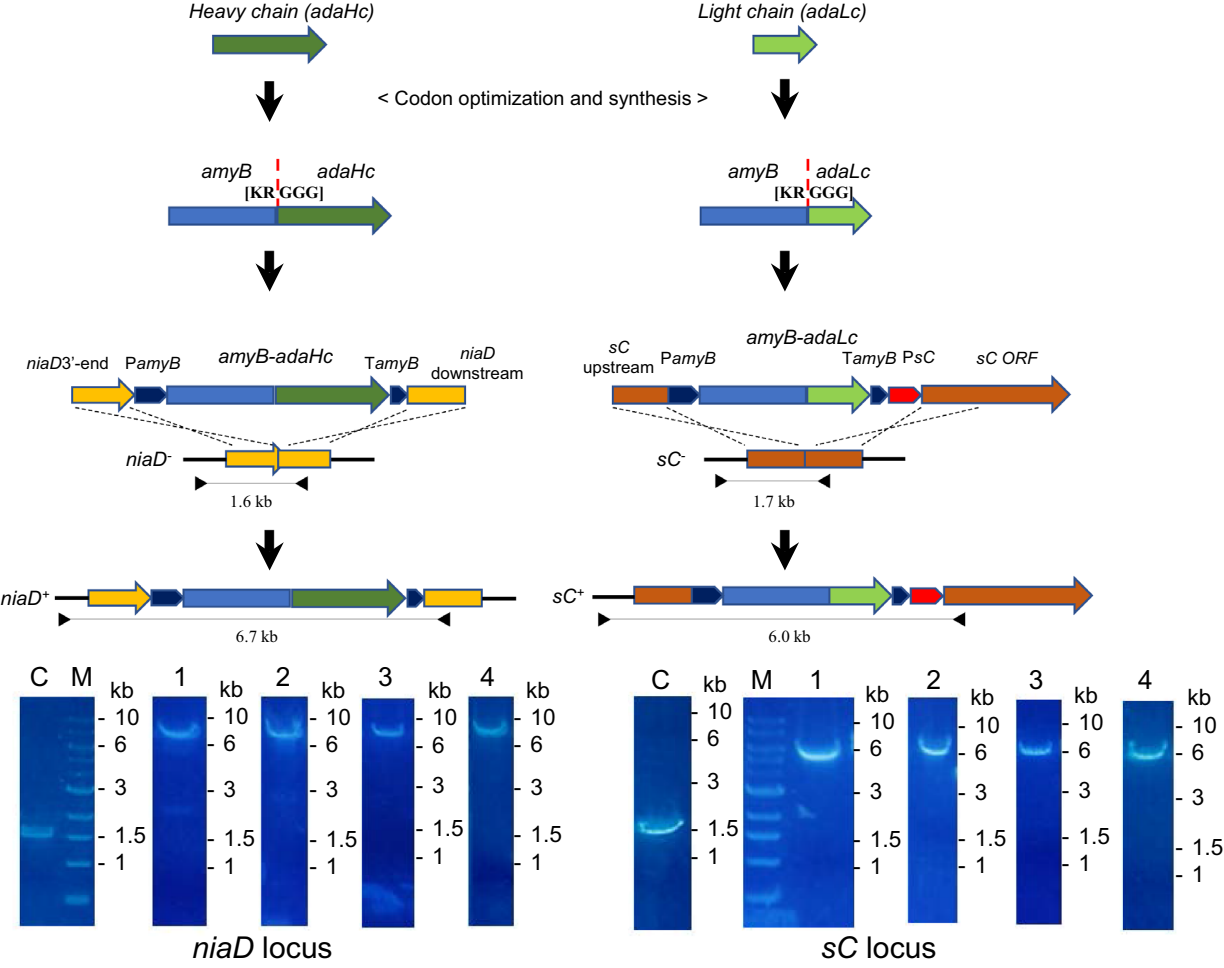
Schematic of the expression construct of adalimumab by *A. oryzae* and the genome PCR. Expression cassettes for heavy chain and light chain of adalimumab were introduced to *niaD* and *sC* loci, respectively. The dashed lines indicate the cleavage site of Kex2-like protease to separate the fusion protein. C, parental strains *niaD*^−^ or *sC*^−^; 1, lD1-ada; 2, lDv10-ada; 3, AUT1-lD-v10-sD-ada; 4, lD-ΔP10-ada; M, DNA marker

**Fig. 2:**
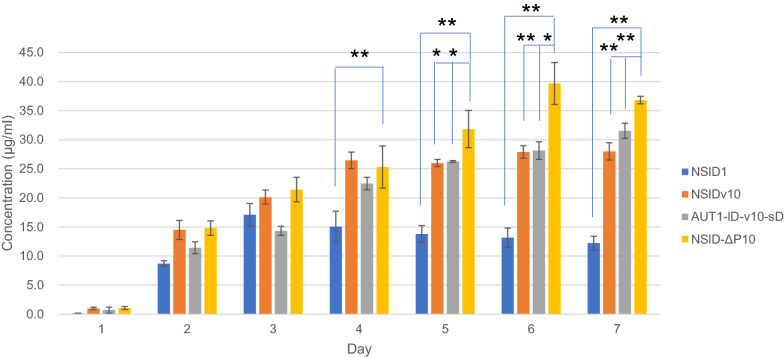
Adalimumab production in the culture supernatant of *A. oryzae*. The producing strains were grown in the 5 × DPY medium. The host strains used for transformation to produce adalimumab were as follows: NSlD1, control strain; NSlDv10, strain with the gene deletion of *Aovps10* for the vacuolar protein sorting receptor; AUT1-lD-v10-sD, hyper-producing mutant (AUT1) strain with double deletion of the tripeptidyl peptidase gene *sedD* and *Aovps10*; NSlD-ΔP10, ten-protease genes deletion strain. Data present the mean of three independent experiments with error bars indicating SD. *, *p* ≤ 0.05; **, *p* ≤ 0.01 (*t* test)

The full-length antibody adalimumab was expressed by *A. oryzae* strains: NSlDv10, AUT1-lD-v10-sD and NSlD-ΔP10, the hyper-producing strains that were successfully used for producing a large amount of foreign proteins in the previous reports. The NSlDv10 strain contains the deletion of *Aovps10* for vacuolar protein sorting receptor to improve the protein secretion by reducing the trafficking pathway from Golgi to vacuoles [32]. In the AUT1-lD-v10-sD strain, beside *Aovps10*, the tripeptidyl peptidase gene *AosedD* was deleted in the hyper-producing mutant (AUT1) strain to further increase the protein production [29]. Additionally, the production loss in the culture medium was avoided by the deletion of ten-protease genes in the NSlD-ΔP10 strain [33]. The production of the full-length antibody requires two oligopeptides of the heavy chain and light chain. They were fused with the α-amylase AmyB and expressed under the control of the *amyB* promoter [30], which is highly induced by culturing in DPY medium. A short sequence coding for KRGGG for the cleavage site of Kex2-like protease was included in the linker to efficiently separate the fusion protein (Fig. 1 and Additional file 1: Fig. S1) as previously performed [30]. For comparison between the strains, codon-optimized genes for heavy chain and light chain of adalimumab (Additional file 1: Fig. S2) were integrated with a single copy into the *niaD* and *sC* loci, respectively (Fig. 1). As expected, the antibody production by these strains was better than the control strain (NSlD1) with the highest accumulation 39.7 mg/L at day 6 in the culture supernatant of the strain of ten-protease genes deletion (Fig. 2). The detectable amount of antibody could be seen after 2 days of incubation. In the control strain, the antibody concentration remained stable or slightly reduced after culturing 3 days, while in other strains the productivity continuously increased until day 6.

**Fig. 3:**
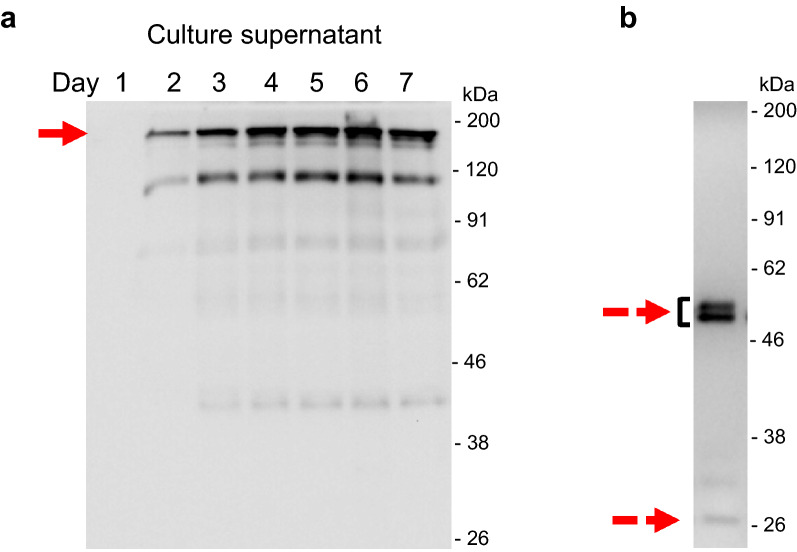
Western blot analysis of adalimumab from *A. oryzae* in the culture supernatant. The NSlD-ΔP10 transformant for producing adalimumab was cultured in 5 × DPY media, and culture supernatant was collected each day for analysis. **a** Time-course analysis in non-reducing condition and **b** the sample of day 6 in reducing condition. The arrow indicates the target IgG, and dashed arrows show the heavy chain and light chain around 50 kDa and 26 kDa, respectively

The appearance of the adalimumab in the culture supernatant was confirmed by Western blot analysis (Fig. 3a). The full-length antibody was resolved by non-reducing SDS-PAGE around 150 kDa. Another major band around 100 kDa might be the heavy-chain dimer, which was also seen in the previous report of *A. awamori* [13]. Moreover, in the reducing condition, the bands of the heavy chain and light chain were observed around 50 kDa and 26 kDa, respectively (Fig. 3b). Two forms of the IgG heavy chain were seen, which may involve the differential *N*-glycosylation, and will be described later. There is no sign of the higher band corresponding to the unprocessed-fusion protein with AmyB protein in the culture supernatant of day 6. Since AmyB has the molecular weight around 55 kDa, the fusion protein of heavy chain and light chain with AmyB protein were anticipated around 105 kDa or 81 kDa, respectively. Therefore, the cleavage between AmyB protein and antibody chains was successful.

### Purification of adalimumab produced by *A. oryzae*

**Fig. 4:**
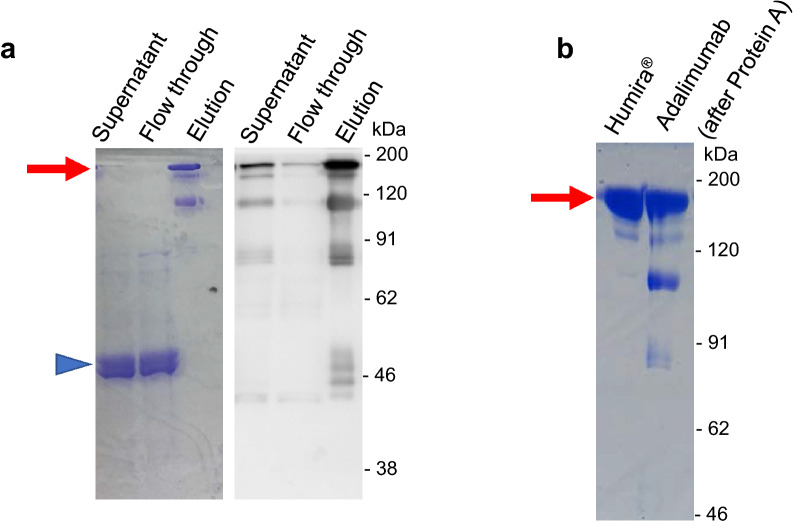
SDS-PAGE analysis of the purified antibody by Protein A affinity chromatography. **a** CBB staining (left) and Western blot (right) analyses and **b** comparison with the commercial product Humira^®^ (AbbVie and Eisai) by CBB staining. The arrow indicates the target antibody around 150 kDa, and the arrowhead shows the AmyB protein

**Fig. 5:**
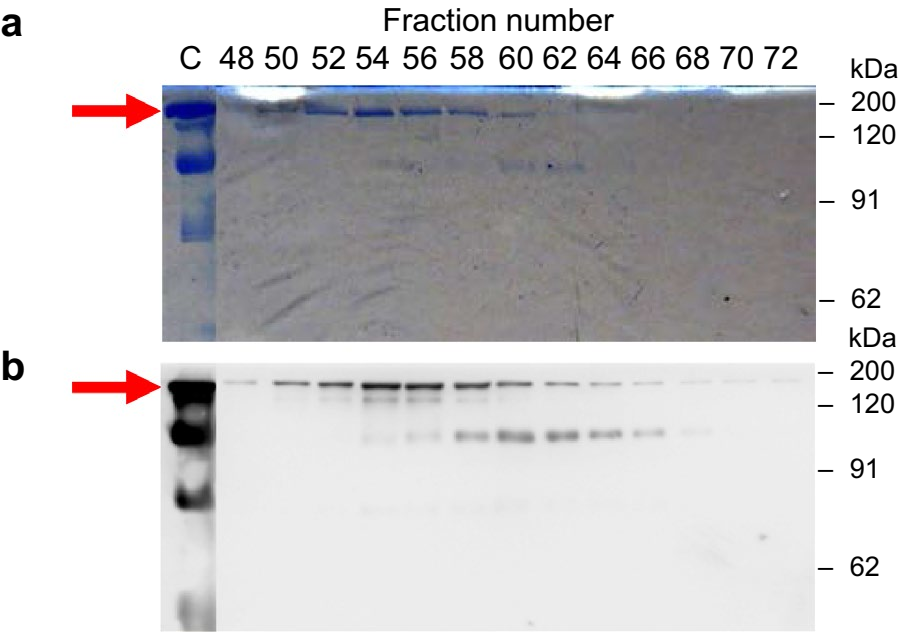
SDS-PAGE of adalimumab from *A. oryzae* by size-exclusion chromatography (SEC) in non-reducing condition. The purified adalimumab by Protein A was applied for secondary purification by SEC to isolate the target antibody at 150 kDa (shown by the arrow). **a** CBB staining and **b** Western blot analysis of the SEC fragments

To determine the characteristics of recombinant adalimumab, Protein A affinity chromatography was applied to capture the antibodies from the culture supernatant. As shown in the Fig. 4a, the target antibody was successfully obtained in the eluted fraction around 150 kDa. However, some minor bands of lower molecular masses were also visible in the purified sample. The comparison with commercial Humira^®^ clearly confirmed the appearance of the target IgG and the unnecessary lower bands (Fig. 4b). The similar results were also reported in case of the antibody trastuzumab produced by *A. awamori*, in which the bands around 100 kDa and 75 kDa were present for a heavy chain dimer and heavy-light chain dimer, respectively [13]. At this point, additional purification was required for obtaining a sample for further analysis, and thus the size-exclusion chromatography (SEC) was selected. The Protein A-purified sample was firstly concentrated to a proper volume using a Vivaspin concentrator (50,000 MWCO). This step can also help to remove other proteins with a molecular weight equal or less than 50 kDa. Then, the concentrated sample was applied to SEC system as described in the Methods. After running through the SEC column, the protein was separated in the range of fractions from 48 to 72 (Fig. 5). As expected, the target antibody was obtained firstly, while other lower molecular weight protein was separated in the later fraction (Fig. 5 and Additional file 1: Fig. S3). The fractions containing the target adalimumab were collected and used in the following experiments.

### Analysis of the *N*-glycosylation in adalimumab produced by *A. oryzae*

**Fig. 6:**
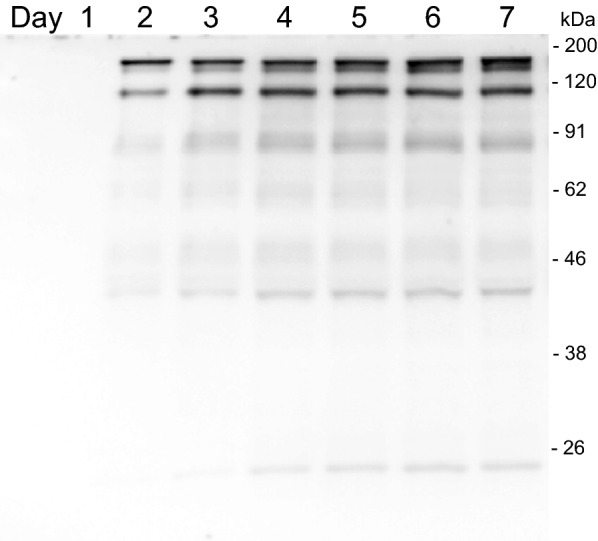
Western blot analysis of adalimumab from the culture supernatant of ∆*Aooch1* strain in non-reducing condition

**Fig. 7:**
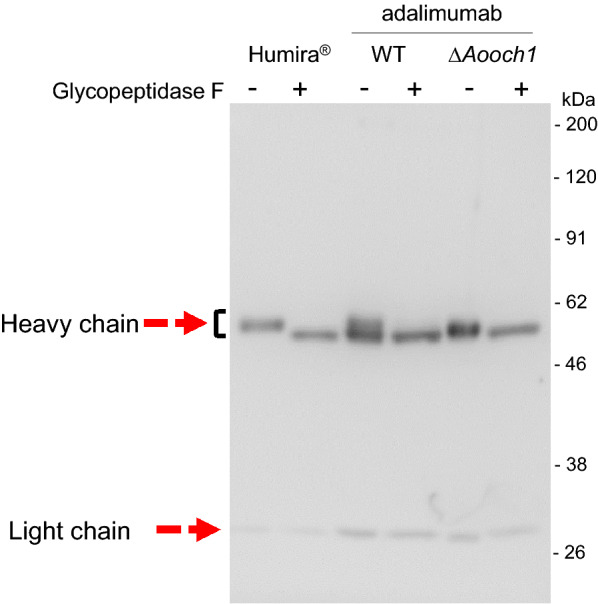
*N*-glycan analysis of adalimumab produced by *A. oryzae* WT and ∆*Aooch1*. Humira^®^ and purified adalimumab produced by *A. oryzae* WT and ∆*Aooch1* with and without Glycopeptidase F treatment were applied to SDS-PAGE for Western blot analysis in reducing condition

**Fig. 8:**
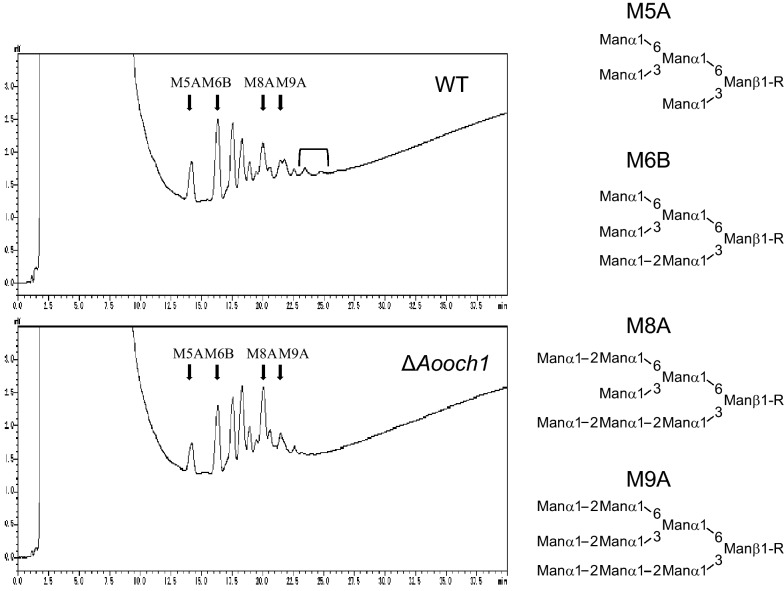
*N*-glycan structure analysis of adalimumab produced by *A. oryzae* WT and ∆*Aooch1*. *N*-glycans were prepared from adalimumab produced by *A. oryzae* WT and ∆*Aooch1* and labeled by 2-aminopyridine (PA). The PA-labelled *N*-glycans were separated by high-performance liquid chromatography (HPLC) both on the Amide-80 column

It is generally accepted that the Och1 plays a crucial role in the divergence of *N*-glycosylation in yeast and filamentous fungi comparing to mammals [34]. In filamentous fungi, its function was demonstrated in *Aspergillus fumigatus* and *Neurospora crassa* [35, 36]. An Och1 ortholog, hereafter referred to as AoOch1, is also found in *A. oryzae* and has the highest identities around 77% with Och1 from *A. fumigatus* (Additional file 1: Fig. S4). Thus, it is expected that AoOch1 also has a similar role in *N*-glycosylation. The *Aooch1* gene was deleted by the CRISPR/Cas9 (Additional file 1: Fig. S5) as described in the Methods. The ∆*Aooch1* strain showed no growth defect or morphological changes (Additional file 1: Fig. S6), which is similar to the report of the *och1* ortholog deletion in *A. fumigatus* [35]. The ∆*Aooch1* strain produced the adalimumab in the culture supernatant as confirmed by Western blot analysis (Fig. 6). Then, the *N*-glycan pattern was analyzed by Western blot analysis. The adalimumab of wild-type (WT) glycosylation displayed two bands of the heavy chain (Fig. 7), a similar result of trastuzumab produced by *A. awamori* in which the lower band presents for non-glycosylation and the upper band indicates the high-mannosylation in the range from 6 to 15 mannose units per mannose-type *N*-glycan [13]. The commercial product Humira^®^ showed only a single band for the heavy chain with a low molecular complex-type *N*-glycan (Fig. 7). In the *A. oryzae* ∆*Aooch1* strain, the adalimumab heavy chain was produced only with a smaller *N*-glycan structure as compared to the high-mannose band of WT, but the unglycosylated heavy chain was not detected (Fig. 7). The different *N*-glycan sizes were confirmed by the cleavage of *N*-glycan with Glycopeptidase F, leading to the same migration in acrylamide gel (Fig. 7). High-performance liquid chromatography (HPLC) analysis revealed a heterogeneity of the *N*-glycan structure in the adalimumab heavy chain, which included Man5GlcNAc2 (M5A), Man6GlcNAc2 (M6B), Man8GlcNAc2 (M8A) and Man9GlcNAc2 (M9A) (Fig. 8). A portion with the molecular weight higher than Man9GlcNAc2 was decreased in the *N*-glycan of ∆*Aooch1* as compared to that of WT (Fig. 8). Collectively, it is suggested that AoOch1 is involved in the generation of high-mannose *N*-glycan in *A. oryzae*.

### Antigen-binding assay by ELISA

**Fig. 9:**
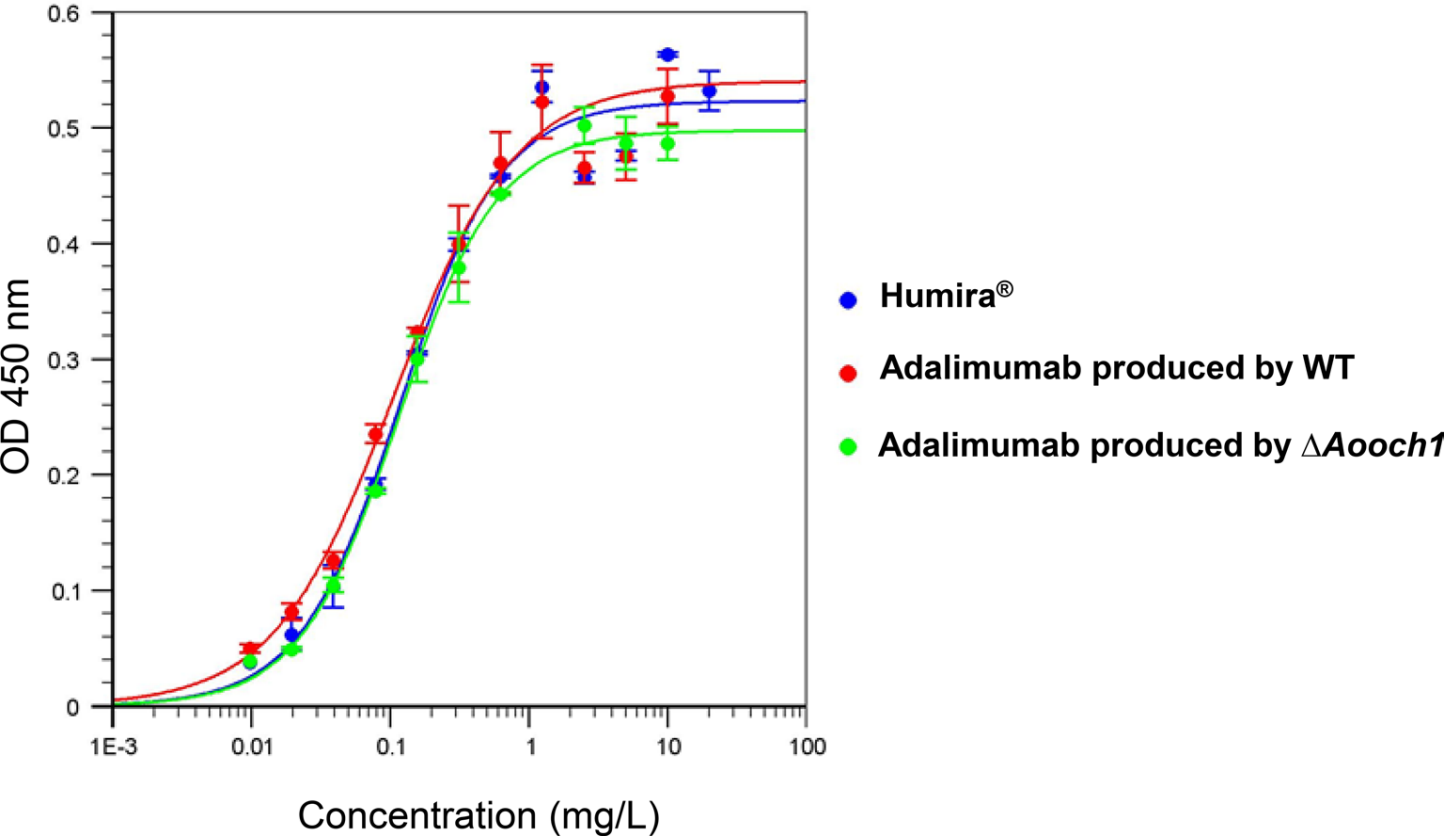
Antigen-binding assay of adalimumab produced by *A. oryzae*. Different concentrations of adalimumab produced by WT and ∆*Aooch1*, and Humira^®^ were incubated in the 96-plate coated with human TNFα. The Anti-IgG (H + L chain) (Human) pAb-HRP antibody was used for detection at OD 450 nm. Each bar represents the standard deviation of the mean. n = 3

The antigen-binding ability of adalimumab produced by *A. oryzae* WT and ∆*Aooch1* strains was tested by ELISA in comparison with Humira^®^. The result showed that no significant difference between the affinity of adalimumab produced in *A. oryzae* and Humira^®^ to the human TNFα (Fig. 9). The similar values of EC50 further confirmed this result at 0.119 mg/L, 0.108 mg/L and 0.095 mg/L with adalimumab produced by *A. oryzae* WT, ∆*Aooch1* and Humira^®^, respectively. Thus, it is concluded that *A. oryzae* can produce the adalimumab with a similar specific antigen-binding activity to that of the commercial product.

### Neutralization of human TNFα-induced cytotoxicity assay

**Fig. 10:**
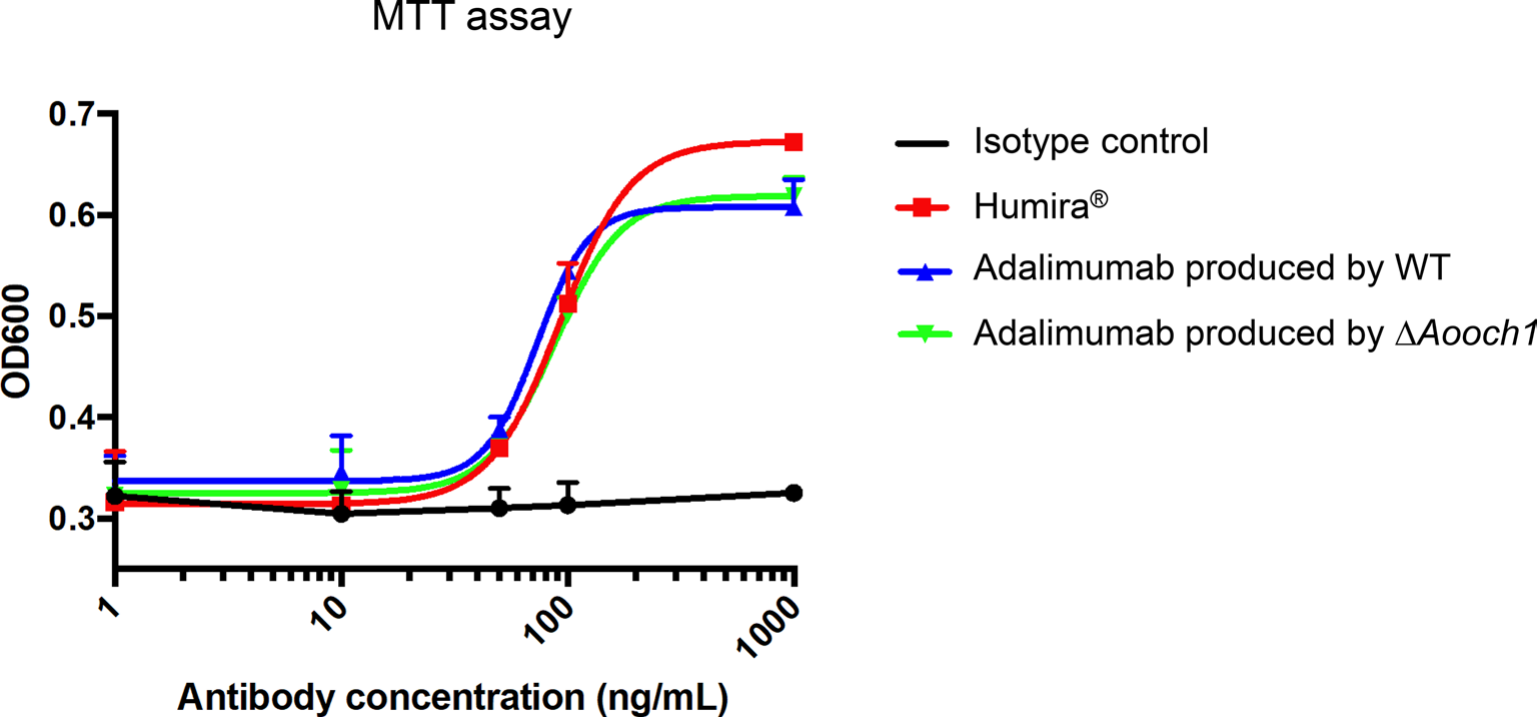
TNFα neutralizing activity of adalimumab produced by *A. oryzae*. For neutralization, 20 ng/mL human TNFα and 1 µg/mL actinomycin D were mixed with different concentration of antibodies. Then, 100 µL of these mixtures were added to the well containing MDA-MB-468 cells. The cell viability was measured by MTT assay at OD 600 nm. Each bar represents the standard deviation of the mean. n = 3

To further confirm the effect of the antigen binding of adalimumab produced by *A. oryzae* toward the living cells, the neutralization activity to inhibit soluble TNFα was investigated and compared to that of the commercial product Humira^®^. Each antibody was co-incubated with 20 ng/mL recombinant human TNFα in a dose-dependent manner and applied to test the MDA-MB-468 cell viability by MTT assay. The graph as shown in Fig. 10 displayed a quite similar efficiency of neutralization between the IgG samples. These results illustrated the level of similarity in bio-functions of adalimumab produced by *A. oryzae* and the approved product Humira^®^ in TNFα-mediated cytotoxicity.

### FcγRIIIa binding assay

**Fig. 11:**
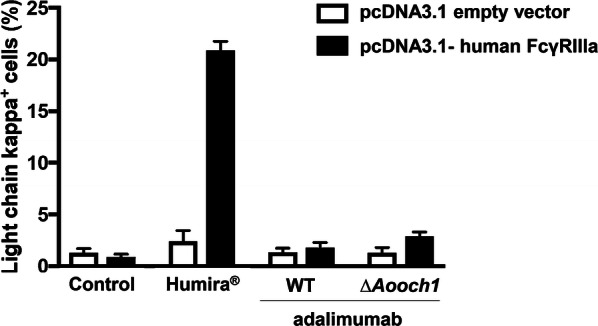
FcγRIIIa binding assay of adalimumab produced by *A. oryzae*. The transfected HEK-292T cell expressing Fc receptor—FcγRIIIa was incubated with adalimumab produced by WT or ∆*Aooch1* (15 µg/mL) mixed 40 ng/mL TNFα. Humira^®^ was used as control. APC anti-Human IgG light chain kappa antibody was used for detection with the absorbance at 405 nm. Each bar represents the standard deviation of the mean. n = 3

Beside the antigen-binding activity, the effector function through the interaction with immune cells at the Fc region is also an important activity to enhance the effectiveness of the therapeutic antibody. Since the *N*-glycan of antibody locates within the interaction zone with Fcγ receptor, the *N*-glycan structure is expected to affect the Fc receptor binding [20]. To examine this issue, FcγRIIIa binding ability of adalimumab produced by WT and ∆*Aooch1* strains were determined in parallel with Humira^®^ (Fig. 11 and Additional file 1: Fig. S7). Although the binding activity was displayed in Humira^®^, adalimumab produced by WT and ∆*Aooch1* did not show any significant binding with FcγRIIIa. This result suggested the *N*-glycan structures containing mannose units but lacking other sugar units such as galactose and *N*-acetylglucosamine could decrease the interaction between adalimumab and FcγRIIIa.

## Discussion

The antibody production by filamentous fungi is less well investigated, as the full-length antibody production has been only reported in trastuzumab by *A. awamori* [13]. In this study, to produce the recombinant antibody for the first time in *A. oryzae*, the heavy and light chains of adalimumab were expressed as the fusion protein with AmyB protein. To select a suitable production strain three genetically modified *A. oryzae* strains, which abundantly produce the heterologous proteins, were tested in this study. Among these hyper-producing strains, the highest amount of adalimumab in the culture supernatant was obtained by NSlD-ΔP10 strain, which features the deletion of ten genes encoding proteases (Fig. 2). The result indicated the major role of the endogenous proteases in the proteolytic degradation of heterologous proteins, especially the complex-structure protein such as antibodies.

The interaction with effector components is required for therapeutic antibody, and most of the approved antibodies are the full-length IgGs [14]. Thus, the full-length IgG is more preferred as a biopharmaceutical. The amount of antibody production is affected by the culture method, and it seems to be quite different among the antibodies even using the same host. Recently, the IgG production by CHO cells can generally reach 5 g/L through optimizing producer cell lines, culture media, and long incubation with high cell densities [37]. In addition, a screening of novel transfected CHO cells from 4345 clones has shown the adalimumab concentration on day 12 ranging from 124 to 594 mg/L by using the batch culture with glucose supplement [38]. In yeast species, the high-throughput screening was applied to select the *P. pastoris* strain producing IgGs up to 1 g/L in a 0.5 L bioreactor [39]. The anti-Her2 IgG was produced by *P. pastoris* with the level of 227 mg/L using 3 L bioreactor [40]. In filamentous fungi, there is one study of trastuzumab production in *A awamori* using 14 L-fermenter with the amount of 900 mg/L [13]. In this study, *A. oryzae* produced the full-length adalimumab with the productivity of 39.7 mg/L in the culture supernatant. Although this may not be directly suitable for industrial application, the antibody productivity by *A. oryzae* can be further improved by several rounds of mutagenesis and additional genetic modifications such as deletion of other protease genes, optimizations involving protein secretion and the increasing of copy-number for heavy and light chains. Moreover, a further enhanced productivity is also expected with the controlled growth environment employing a bioreactor as previously reported in *A. oryzae* [41], which will meet the industrially competitive level.

The full-length antibody normally contains two *N*-glycans on the Fc region of pair heavy chains at Asn297 [42], and adalimumab also has the *N*-glycosylation sites in Fc region at Asn301 in the sequence Asn-Ser-Thr of the typical motif (Asn)-X-Ser/Thr (where X is any amino acid except a Pro) [43]. The two bands of adalimumab heavy chain produced by *A. oryzae* WT strain in Fig. 7 may correspond to high mannose-type glycan (more than or around 9 mannose units) and non-glycosylation (lower) as reported in *A. awamori* [13]. The complex-type glycan in Humira^®^ with a low molecular weight placed the corresponding band in the middle position between the two bands of adalimumab produced by *A. oryzae* WT (Fig. 7). In the Δ*Aooch1*, only a single band was found at a lower position of the upper band of WT (Fig. 7), which suggests the ratio reductions of the heavy chain with high mannose-type glycan. In HPLC analysis, a heterogeneous profile of the *N*-glycan structure was obtained in adalimumab heavy chain produced by *A. oryzae* (Fig. 8). However, a smaller number of mannoses (mainly from Man5GlcNAc2 to Man9GlcNAc2) in the *N*-glycan from *A. oryzae* WT were detected than in those from the secreted proteins of yeasts *S. cerevisiae* and *P. pastoris* [25, 44], and therefore a contribution of Och1-mediated high-mannosylation is present but small in *A. oryzae*.

One of the most important activities of the IgG is the binding ability to the target antigen. It is suggested that the *N*-glycan structure does not affect the antigen affinity due to the far distance between the *N*-glycosylation site and the Fv domain [45]. Hence, in case of adalimumab produced by *A. oryzae*, the antigen-binding affinity with TNFα was expected to be equivalent between the WT and ∆*Aooch1* samples. In the antigen affinity measurement (Fig. 9), both adalimumab produced by WT and ∆*Aooch1* exhibited the binding activity with a similar level to that of the commercial product Humira^®^. Moreover, the ability to neutralize cell toxicity mediated by TNFα also confirmed the high similarity of their bio-functional activity (Fig. 10), which clearly demonstrates the potential of *A. oryzae* in the functional antibody production.

Fcγ receptors play critical roles in phagocytosis, endocytosis and antibody-dependent cellular cytotoxicity (ADCC) [46]. One of the receptors, FcγRIIIa, plays a significant role in the resistance to infection and cancer by activating the immune cells especially natural killer (NK) cells, in which immune complexes with Fc region of antibody induce ADCC [47]. The conformation of the Fc region is changed after the antigen binding, which influences the effector function of IgG [48]. Formation of antibody-antigen complex recruits adalimumab and stimulates its interaction possibility with FcγRIIIa [49]. The mutation abrogating the *N*-glycosylation at Asn in the Fc region aborts the interaction with FcγRIIIa [50]. The fucosylated antibody was reported to reduce the binding affinity to FcγRIIIa [51]. In Humira^®^, the fucosylated *N*-glycan is contained with the ratio of 10% [52], and thus relatively low affinity to FcγRIIIa was detected when high concentrations of Humira^®^ (15 µg/mL) and TNFα (40 ng/mL) were used (Fig. 11). In contrast, no apparent binding was found with the adalimumab produced by *A. oryzae*, and the *N*-glycan alteration by *Aooch1* deletion did not improve the FcγRIIIa binding (Fig. 11). Based on the crystal structure of the *N*-glycan attached to Asn297 of IgG Fc region together with FcγRIIIa (PDB entry code 4CDH), the *N*-glycan is close to the binding surface of FcγRIIIa [45]; alteration in the *N*-glycan structure involving ion charge and steric allocation would shift the Fc region-FcγRIIIa interaction to the inappropriate position. On the other hand, the bulky mannose structure of *N*-glycan in adalimumab produced by *A. oryzae* WT strain may interfere the approach of FcγRIIIa to bind with the Fc region due to stereometric occupation. The previous study demonstrated that the mannose-type glycan of rituximab produced by *P. pastoris* has a higher FcγRIIIa affinity as compared to the fucosylated complex-type glycan from CHO cell line [53]. The similar comparative analysis revealed the highest FcγRIIIa affinity with the afucosylated complex-type glycan [54], and thus this enhancement is likely due to the afucosylated glycan [55]. In case of the adalimumab, the afucosylated complex-type glycan of Humira^®^ could have a dominant effect on the FcγRIIIa binding, while the mannnose-type glycan of adalimumab produced by *A. oryzae* WT and *Aooch1* deletion strains are inadequate. In addition, the adalimumab of Humira^®^ shows some FcγRIIIa binding activity to lyse the TNF-expressing cell in ADCC test; but the mechanism has not yet been clarified and may differ from the response to various TNF-related diseases [49]. Hence, further research is required to determine the effect of the *N*-glycan structure on the binding activity between the adalimumab and the Fc receptor.

A biosimilar product to one of the world’s best-selling IgGs—Humira^®^—was generated by *A. oryzae* in this study. The full-length of adalimumab was obtained with the equivalent abilities in antigen binding and neutralization. The long history of food and research has provided a strong background for *A. oryzae* in optimized cultivation and genetic manipulation [56]. These features would strengthen the industrial application of *A. oryzae* as a low-cost platform for not only adalimumab or other IgGs but also novel biopharmaceutical products in diagnostic and therapy. In adalimumab, the ADCC activity corresponding to Fc receptor binding has not been established clearly yet [57]. To avoid any possible impact and support other bio-pharmaceutical production, mimicking the mammalian *N*-glycan structure will be necessary in *A. oryzae*. The *N*-glycosylation modification in filamentous fungi is still in the initial step to produce the glycan core, which needs to be catalyzed by additional enzymes for converting to the mammalian-like glycan structure [22]. The effect of the *Aooch1* gene on the *N*-glycan structure and bio-function of adalimumab from *A. oryzae* was demonstrated in this study. To achieve the ultimate goal of mimicking the mammalian *N*-glycan structure, CRISPR/Cas9 system would be used to knock-out the genes in the high-mannose pathway and to introduce the genes for adding correct sugar monomer to the glycan core-structure, which may be considered from the glycan-engineering process in *P. pastoris* [27]. While this study identified the primary roles of adalimumab produced by *A. oryzae* in antigen biding and neutralization, for biopharmaceutical application it will be required to define the quality attributes by detailed biological and structural characterizations as previously reported [52, 58]. Further qualitative and quantitative improvement in *A. oryzae* as a host would lead to the establishment of a promising production platform of biopharmaceutical products.

## Conclusion

The great demand together with the requirement for reducing cost of antibodies has drawn more attention to finding a suitable expression platform for producing recombinant antibodies. Our study demonstrated that adalimumab can be successfully produced in the culture supernatant of *A. oryzae* transformants with similar affinities and biological activities to its commercial form. This study will stimulate the application of *A. oryzae* or filamentous fungi, in general, to be used for the industrial production of pharmaceutical proteins.

## Methods

### Materials and chemical

Humira^®^—AbbVie and Eisai.

### Strains and growth media

*Escherichia coli* DH5α (Takara Bio, Shiga, Japan) was cultured in LB medium (1% peptone, 0.5% yeast extract, and 0.5% NaCl). The *A. oryzae* wild-type strain, RIB40 [59] and a strain with a highly efficient gene-targeting background (*niaD*^−^*sC*^−^ ∆*ligD*), NSlD1 [32], were used as a DNA donor. The *A. oryzae* strains used in this study for antibody production are listed in the Table 1. The conidia of *A. oryzae* were collected by growth on the PDA agar medium (Potato Dextrose Agar; Nissui Pharmaceutical, Tokyo, Japan). DPY medium containing 2% dextrin, 1% polypeptone, 0.5% yeast extract, 0.5% KH_2_PO_4_, and 0.05% MgSO_4_·7H_2_O was used for the pre-culture of transformants. Czapek-Dox (CD) medium (2% glucose, 0.3% NaNO_3_, 0.2% KCl, 0.1% KH_2_PO_4_, 0.05% MgSO_4_·7H_2_O, and 0.002% FeSO_4_.7H_2_O, pH 5.5) was used for selection using *niaD* and *sC*-based plasmid integration. To produce antibody, 5 × DPY medium containing 10% dextrin, 5% polypeptone, 2.5% yeast extract, 0.5% K_2_HPO_4_, and 0.05% MgSO_4_·7H_2_O was used.

**Table 1:** Strains used in this study

Stain name	Genotype	References
RIB40	Wild-type	[59]
NSlD1	*niaD*^−^*sC*^−^*adeA*^−^*∆argB*::*adeA*^−^*∆ligD*::*argB ∆pyrG*::*adeA*	[32]
NSlDv10	*niaD*^−^*sC*^−^*adeA*^−^*∆argB*::*adeA*^−^*∆ligD*::*argB ∆pyrG*::*adeA ∆Aovps10*::*pyrG*	[32]
AUT1-lD-v10-sD	*niaD*^−^*sC*^−^*adeA*^−^ Δ*argB*::*adeA*^−^ Δ*tppA*::*argB* Δ*pepE*::adeA *aut1*^−^ Δ*ligD* Δ*pyrG* Δ*Aovps10* Δ*AosedD*::*pyrG*	[29]
NSlD-ΔP10	*niaD*^−^*sC*^−^*adeA*^−^ Δ*argB*::*adeA*^−^ Δ*ligD*::*argB* Δ*pyrG*::*adeA* Δ*tppA* Δ*pepE* Δ*nptB* Δ*dppIV* Δ*dppV* Δ*alpA* Δ*pepA* Δ*AopepAa* Δ*AopepAd* Δ*cpI*::*pyrG*	[33]
lD1-ada	*niaD*^−^::[P*amyB*::*amyB*-*(KRGGG)*-*adaHc*::T*amyB*::*niaD*] *sC*^−^::[P*amyB*::*amyB*-*(KRGGG)*-*adaLc::*T*amyB::sC*] *adeA*^−^*∆argB*::*adeA*^−^*∆ligD*::*argB ∆pyrG*::*adeA*	This study
lDv10-ada	*niaD*^−^::[P*amyB*::*amyB*-*(KRGGG)*-*adaHc*::T*amyB*::*niaD*] *sC*^−^::[P*amyB*::*amyB*-*(KRGGG)*-*adaLc*::T*amyB*::*sC*] *adeA*^−^*∆argB*::*adeA*^−^*∆ligD*::*argB ∆pyrG*::*adeA* *∆Aovps10*::*pyrG*	This study
AUT1-lD-v10-sD-ada	*niaD*^−^::[P*amyB*::*amyB*-*(KRGGG)*-*adaHc*::T*amyB*::*niaD*] *sC*^−^::[P*amyB*::*amyB*-*(KRGGG)*-*adaLc*::T*amyB*::*sC*] *adeA*^−^ Δ*argB*::*adeA*^−^ Δ*tppA*::*argB* Δ*pepE*::*adeA* *aut1*^−^ Δ*ligD* Δ*pyrG* Δ*Aovps10* Δ*AosedD*::*pyrG*	This study
lD-ΔP10-ada	*niaD*^−^::[P*amyB*::*amyB*-*(KRGGG)*-*adaHc*::T*amyB*::*niaD*] *sC*^−^::[P*amyB*::*amyB*-*(KRGGG)*-*adaLc*::T*amyB*::*sC*] *adeA*^−^ Δ*argB*::*adeA*^−^ Δ*ligD*::*argB* Δ*pyrG*::*adeA* Δ*tppA* Δ*pepE* Δ*nptB* Δ*dppIV* Δ*dppV* Δ*alpA* Δ*pepA* Δ*AopepAa* Δ*AopepAd* Δ*cpI*::*pyrG*	This study
lD-ΔP10-ada-Δaooch1	*niaD*^−^::[P*amyB::amyB*-*(KRGGG)*-*adaHc*::T*amyB*::*niaD*] *sC*^−^::[P*amyB*::*amyB*-*(KRGGG)*-*adaLc*::T*amyB*::*sC*] *adeA*^−^ Δ*argB*::*adeA*^−^ Δ*ligD*::*argB* Δ*pyrG*::*adeA* Δ*tppA* Δ*pepE* Δ*nptB* Δ*dppIV* Δ*dppV* Δ*alpA* Δ*pepA* Δ*AopepAa* Δ*AopepAd* Δ*cpI*::*pyrG* Δ*Aooch1*	This study

### Plasmid construction

First, the DNA sequences of heavy chain and light chain of adalimumab were obtained from the Drugbank database (https://www.drugbank.ca/) and optimized by the codon usage table of *A. oryzae*. Then, they were synthesized by GeneArt Gene Synthesis (Thermo Fisher Scientific, Waltham, MA, USA). The open reading frame encoded a fusion protein consisting of the α-amylase gene (*amyB*), a short linker including the sequence encoding for KRGGG (cleavage site for Kex2-like protease) and the mature heavy or light chain of adalimumab. Vectors pUtNAN [60] and pisCIIA [61] were used for the transformation of *A. oryzae* to introduce expression cassettes containing adalimumab’s heavy chain or light chain into the *niaD* and *sC* loci, respectively. These vectors contain the dextrin-inducible *amyB* promoter and *amyB* terminator, and the expression cassette was inserted at the *Sma*I site located at the downstream of the *amyB* promoter.

### Transformation and expression in *A. oryzae*

Transformation of *A. oryzae* was performed as described previously [62], and transformants were selected by growth on CD minimal agar medium. Transformants were transferred to a new selective medium twice, and the colony PCR using KOD FX Neo DNA polymerase (Toyobo, Tokyo, Japan) was applied to confirm the correct transformants.

To induce antibody production, the conidia of *A. oryzae* transformants were inoculated in 5 × DPY liquid medium (pH 8.0) with 1 × 10^7^ conidia per 100 mL. After incubation at 30 °C for 1–7 days at 150 rpm, the culture supernatant was collected by filtrating with Miracloth for further analysis.

### Quantification of antibody

The IgG concentration in the sample was measured by standard ELISA process using goat anti-Human IgG (Southern Biotech, Birmingham, AL, USA) for the capture step. The standard curve was built with the Human IgG isotype control (Genway, San Diago, CA, USA). Goat anti-Human IgG-AP (Southern Biotech) was used for IgG detection. The results were read at the absorbance 405 nm by using TriStar2 LB942 Multimode Reader (Berthold Technologies, Bad Wildbad, Germany).

### Antibody detection

The sample was mixed with an approximate volume of 5 × sample loading buffer [250 mM Tris–HCl (pH 6.8), 10% SDS, 50% glycerol, 0.025% bromophenol blue, 250 mM dithiothreitol (DTT); without DTT in case of non-reducing condition) and subjected to sodium dodecyl sulfate–polyacrylamide gel electrophoresis (SDS-PAGE). Either the gels were stained for protein with Coomassie Brilliant Blue (Nacalai, Kyoto, Japan) or the proteins were transferred to membrane Immobilon-P membranes (0.45 µm; Merck Millipore, Tokyo, Japan) for Western blotting. Anti-IgG (H + L chain) (Human) pAb-HRP (Medical & Biological Laboratories CO., LTD, Nagoya, Japan) was used for detection by Western Lightning Plus system (PerkinElmer, Waltham, MA, USA). The target protein bands were visualized through luminescent image analyzer (LAS-4000 mini; Fujifilm, Tokyo, Japan).

### Purification

The supernatant of *A. oryzae* transformant was applied to Protein A Sepharose™ 4 Fast Flow system (GE Healthcare, Chicago, IL, USA) at room temperature. The antibody purification was performed by the instruction of the manufacturer with phosphate-buffered saline (PBS) as the equilibrating and washing buffers. The target protein was eluted with 0.1 M citric acid (pH 3.5), and 1 M Tris–HCl (pH 9.0) was quickly added to neutralize the eluted fraction for preserving the antibody activity.

To achieve higher purity, the antibody sample was subsequently performed by size-exclusion chromatography (SEC). Firstly, the Protein A-purified sample was collected and concentrated to proper volume by using Vivaspin Turbo ultrafiltration spin column 50 K (Sartorius Lab, Göttingen, Germany). Secondly, the concentrated sample was loaded to the HiLoad™ 26/60 Superdex™ 200 prep grade (GE Healthcare) in the ÄKTA purifier chromatography system at 4 °C. The flow rate was maintained at 2 mL/min with PBS buffer (pH 7.4).

### Deletion of *Aooch1* gene in *A. oryzae* by CRISPR/Cas9 system

To delete the target gene, the genome-editing plasmid together with a circular donor DNA plasmid was generated as described by Katayama et al. [60]. The sequence GTGGTTCCAGACGACACCCA in the middle of *Aooch1* gene was selected to make the sgRNA cassette with *U6* promotor. The genome-editing plasmid was created by introducing the sgRNA expression cassette to the pRGE-gRT6 plasmid [60] at the *Sma*I cutting site. Meanwhile, the 1 kb fragments of upstream and downstream of *Aooch1* gene (gene ID AO090120000208) were amplified and joined together. The flanking sequences were inserted into the pUC19 linearized vector (Takara Bio) to create the donor plasmid. Both of the plasmids were applied to transformation using the NSlD-ΔP10-derived strain producing adalimumab as mentioned above.

### *N*-glycan analysis

For Glycopeptidase F (Takara Bio) treatment, 10 μl of protein sample was mixed with 2.5 μl of Denatured buffer with 0.2 M 2-mercaptoethanol and heated at 100 °C for 3 min. Stabilizer solution (5 μl) was added and then mixed with 5.5 μl of distilled water. The reaction was carried by adding 2 μl (1 mU) of Glycopeptidase F and incubating at 37 °C for 15–20 h.

After SDS-PAGE, the gel containing the heavy chain was excised and subjected to in-gel digestion with trypsin. The generated peptides were extracted and incubated with Peptide-*N*-Glycosidase F (New English BioLabs, Beverly, MA, USA) at 37 °C for 16 h. The released *N*-glycans were purified and labelled with 2-aminopyridine (PA) using BlotGlyco (Sumitomo Bakelite, Tokyo, Japan) according to the manufacturer’s instructions. The PA-labeled *N*-glycan was analyzed by high-performance liquid chromatography (HPLC) using a size-fractionation column (TSKgel Amide-80, 4.6 × 250 mm, Tosoh, Tokyo, Japan) according to the method described by Chiba et al. [24].

### Enzyme-linked immunosorbent assay (ELISA)

The binding activity of antibody to its antigen, recombinant human TNFα (BioLegend, San Diego, CA, USA), was measured by using 96-well Nunc MaxiSorp™ Flat-Bottom plate (Thermo Fisher Scientific). First, 100 μl TNFα solution was coated with a concentration of 100 ng/mL in sodium carbonate buffer (pH 9.6) overnight at 4  °C. The wells were washed three times with PBS-Tween solution (PBS buffer with 0.05% Tween 20) and blocked with blocking buffer (PBS-Tween containing 5% skim milk) at room temperature for 1 h. After washing three times with the PBS-Tween solution, 100 μl of adalimumab sample in serial dilution was added to each well and incubated at room temperature for 1 h. Then, the wells were washed again three times with PBS-Tween solution and incubated with 1:8000-diluted Anti-IgG (H + L chain) (Human) pAb-HRP antibody at room temperature for 1 h. The plate was washed four times with PBS-Tween solution and incubated with 100 μl ELISA POD Substrate TMB Solution (Nacalai) at room temperature. The reaction was stopped by adding an equal volume of 1 M H_2_SO_4_, and the absorbance was measured at 450 nm using Multiskan FC microplate reader (Thermo Fisher Scientific).

### Neutralization of human TNFα-induced cytotoxicity assay

The MDA-MB-468 cells were cultured into 96-well plate at a 5 × 10^4^ cells/well density in D-MEM/Ham’s F-12 media (Fujifilm Wako, Osaka, Japan) supplemented with 10% fetal bovine serum (Sigma Aldrich, St Louis, MO, USA), 50 µg/mL gentamycin (Nacalai), 0.1 µM 2-mercaptoethanol (Nacalai), 100 ng/mL actinomycin D (Sigma) and 20 ng/mL human TNFα (BioLegend). Subsequently, cells were incubated with three different types of anti-human TNFα antibody or human IgG isotype antibody in the range from 1 to 1000 ng/mL concentrations. After 24 h, cell viability was analyzed using the Cell Proliferation Kit I (Roche, Mannheim, Germany). Briefly, 0.5 mg/mL methylthiazolyldiphenyl-tetrazolium bromide (MTT) labeling reagent was added in cell culture media. After 3 h, 100 µL of the solubilization solution was added into each well, and the culture plate was incubated for 24 h at 37 °C. The absorbance of the plate was measured at 600 nm by TriStar2 LB942 Multimode Reader (Berthold Technologies).

### FcγRIIIa binding assay

The human FcγRIIIa coding sequence was purchased from RIKEN Human cDNA Clones in Japan (clone ID: 5180561). The gene ORF sequence was amplified by PCR and cloned into the pcDNA3.1(+) expression vector. HEK-293T cells were cultured into 12-well plate in D-MEM/Ham’s F-12 media supplemented with 10% fetal bovine serum, 50 µg/mL gentamycin and 0.1 µM 2-mercaptoethanol until 70–80% confluence. The complex of 10 µg Polyethylenimine (Sigma) and 1.5 µg of pcDNA3.1- human FcγRIIIa or pcDNA3.1 empty vector in serum-free media was gently added into cell culture media. After 24 h, expression of human FcγRIIIa was confirmed by staining of the PE anti-human FcγRIIIa antibody (BioLegend) in the SA3800 Spectral Analyzer (Sony Biotechnology, San Jose, CA, USA). After aspiration of culture media of transfected cells, three different types of 15 µg/mL anti-human TNFα antibody and 40 ng/mL recombinant human TNFα in serum-free D-MEM/Ham’s F-12 media were added into cells. At 3 h after incubation, these cells were harvested and stained by the APC anti-human light chain kappa antibody (BioLegend). Antibody-binding cells were detected by the SA3800 Spectral Analyzer.

## Supplementary information

**Additional file 1.** Additional figures.

## Data Availability

All data supporting the results are included within this article and its additional file. Plasmids and strains are available upon request.

## Abbreviations

Fab: Fragment antigen-binding
Fc: Fragment crystallizable region
CHO: Chinese hamster ovary
ADCC: Antibody-dependent cell-mediated cytotoxicity
GRAS: Generally Regarded As Safe
CRISPR: Clustered Regularly Interspaced Short Palindromic Repeats
