# Supplementary material for: Functional production of human antibody by the filamentous fungus Aspergillus oryzae
Source: Fungal Biol Biotechnol. 2020 May 28;7:7. doi: 10.1186/s40694-020-00098-w (PMC7257131; doi:10.1186/s40694-020-00098-w)
Supplement: Supplementary file 1 — Additional file 1. Additional figures. [file 40694_2020_98_MOESM1_ESM.pdf]

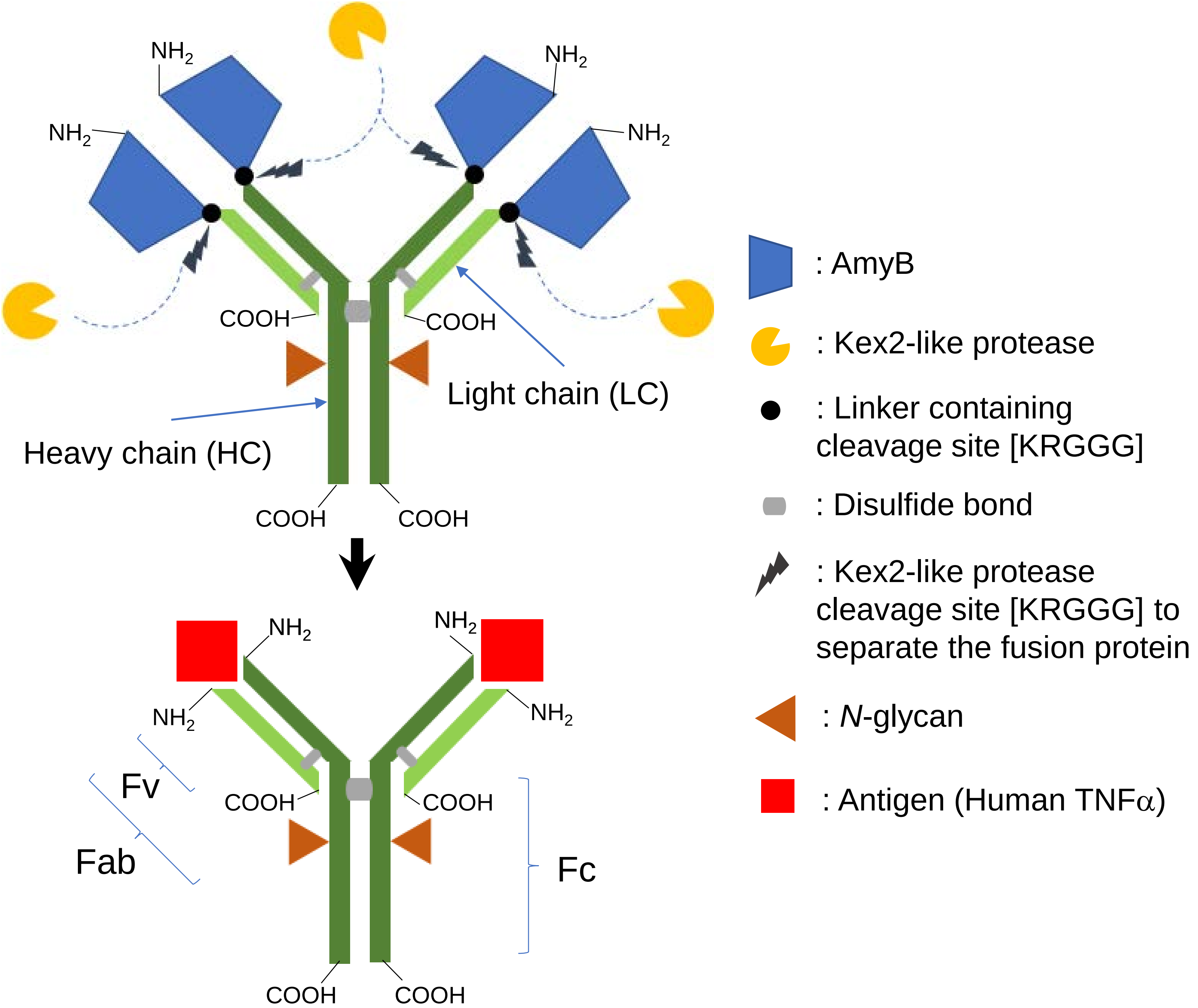

Fig. S1. Schematic structure of fusion protein and full-length adalimumab produced by *A. oryzae*

Codon-optimized sequence for adalimumab (based on the *A. oryzae tef1* sequence)

Heavy chain:

GAGGTTCAGCTTGTCGAGTCTGGTGGTGGTCTTGTCCAGCCCGGTCGTTCTCTTCGTCTCTCCT
GCGCTGCCTCCGGTTTCACCTTCGATGACTACGCCATGCACTGGGTCCGTCAGGCCCCTGGTA
AGGGTCTTGAATGGGTCTCTGCCATCACCTGGAACTCTGGTCACATCGATTACGCTGACTCCGT
TGAGGGTCGTTTCACCATCTCTCGTGACAACGCCAAGAACTCCCTTTACCTCCAGATGAACTCT
CTCCGTGCTGAGGATACTGCTGTCTACTACTGCGCTAAGGTCTCCTACCTCTCCACTGCCTCTTC
TCTTGACTACTGGGGTCAGGGTACCCTCGTTACCGTCTCCTCTGCTTCCACCAAGGGTCCCTCT
GTCTTCCCTCTCGCTCCCTCCTCCAAGTCCACCTCCGGAGGTACCGCTGCCCTTGGTTGTCTCG
TTAAGGATTACTTCCCTGAGCCCGTCACTGTCTCCTGGAACTCTGGTGCCCTCACCTCCGGTGT
CCACACCTTCCCCGCTGTCCTCCAGTCCTCTGGTCTCTACTCCCTCTCTTCCGTTGTCACCGTTC
CTTCTTCCTCTCTCGGTACTCAGACCTACATCTGCAACGTCAACCACAAGCCTTCCAACACTAA
GGTCGACAAGAAGGTTGAGCCCAAGTCTTGCGATAAGACTCACACTTGCCCTCCCTGCCCTGC
TCCCGAGCTTCTCGGTGGTCCCTCCGTTTTCCTGTTCCCTCCTAAGCCCAAGGACACCCTTATG
ATTTCCCGTACTCCTGAAGTCACCTGCGTCGTTGTCGATGTCTCCCACGAGGATCCCGAAGTCA
AGTTCAACTGGTACGTCGACGGCGTTGAGGTCCACAACGCCAAGACCAAGCCCCGCGAGGA
GCAGTACAACTCCACTTACCGTGTCGTTTCCGTTCTTACTGTTCTTCACCAGGATTGGCTCAAC
GGTAAGGAATACAAGTGCAAGGTCTCCAACAAGGCCCTTCCCGCTCCTATCGAGAAGACCATC
TCCAAGGCTAAGGGTCAGCCTCGTGAGCCTCAGGTCTACACTCTTCCTCCCTCTCGTGACGAG
CTCACCAAGAACCAGGTCTCCCTTACTTGCCTCGTCAAGGGTTTCTACCCCTCCGACATCGCCG
TCGAGTGGGAGTCCAACGGTCAGCCCGAGAACAACTACAAGACCACTCCTCCCGTCCTTGAC
TCCGACGGTTCCTTCTTCCTCTACTCCAAGCTCACTGTTGACAAGTCTCGTTGGCAGCAGGGC
AACGTCTTCTCCTGCTCCGTCATGCACGAGGCCCTCCACAACCACTACACTCAGAAGTCTCTTT
CCCTCTCTCCTGGTAAGTAA

Light chain:

GATATCCAGATGACCCAGTCCCCTTCTTCCCTTTCTGCCTCCGTTGGTGACCGTGTCACCATCA
CCTGCCGTGCCTCTCAGGGCATTCGTAACTACCTTGCCTGGTACCAGCAGAAGCCCGGTAAG
GCTCCTAAGCTCCTCATCTACGCCGCCTCCACTCTTCAGTCTGGTGTCCCTTCCCGTTTCTCCG
GTTCCGGTTCTGGTACTGACTTCACCCTCACCATCTCCTCCCTTCAGCCCGAAGATGTTGCTA
CTTACTACTGCCAGCGTTACAACCGCGCTCCTTACACTTTCGGTCAGGGTACCAAGGTCGAG
ATCAAGCGTACCGTTGCCGCTCCCTCCGTCTTCATCTTCCCTCCCTCTGACGAGCAGCTCAAG
TCCGGTACCGCTTCCGTTGTCTGCCTCCTCAACAACTTCTACCCTCGTGAGGCTAAGGTCCAG
TGGAAGGTCGACAACGCCCTCCAGTCCGGTAACTCTCAGGAGTCCGTTACCGAGCAGGATT
CTAAGGACTCCACCTACTCTCTTTCCTCCACTCTCACCCTTTCCAAGGCTGACTACGAGAAGC
ACAAGGTCTACGCTTGCGAAGTCACCCACCAGGGTCTCTCCTCTCCTGTTACCAAGTCCTTCA
ACCGTGGTGAGTGCTAA

Fig. S2. Nucleotide sequence of genes encoding adalimumab heavy and light chains after codon optimization

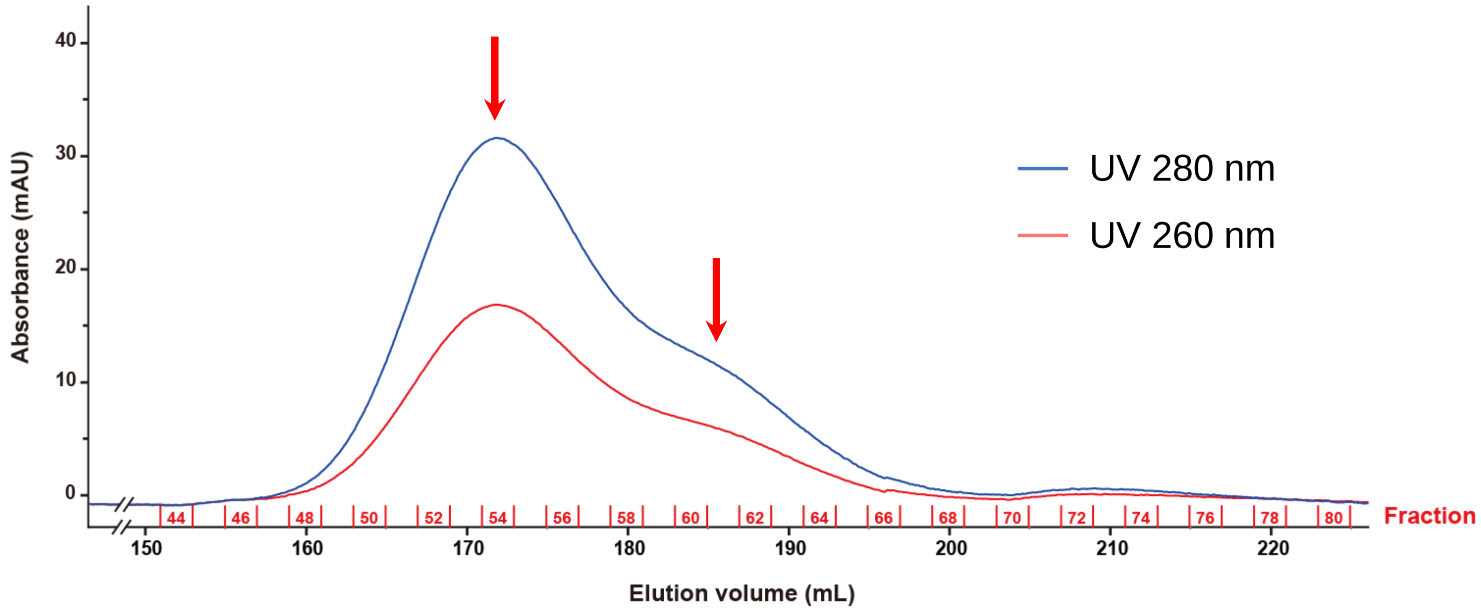

Fig. S3. Chromatogram of SEC purification by using HiLoad™ 26/600 Superdex™ 200 prep grade column, generated by UNICORN software (GE Healthcare Life Sciences). The arrows indicated two peaks of the separated protein by SEC, corresponding to the target antibody band around 150 kDa and the lower protein band around 100 kDa.

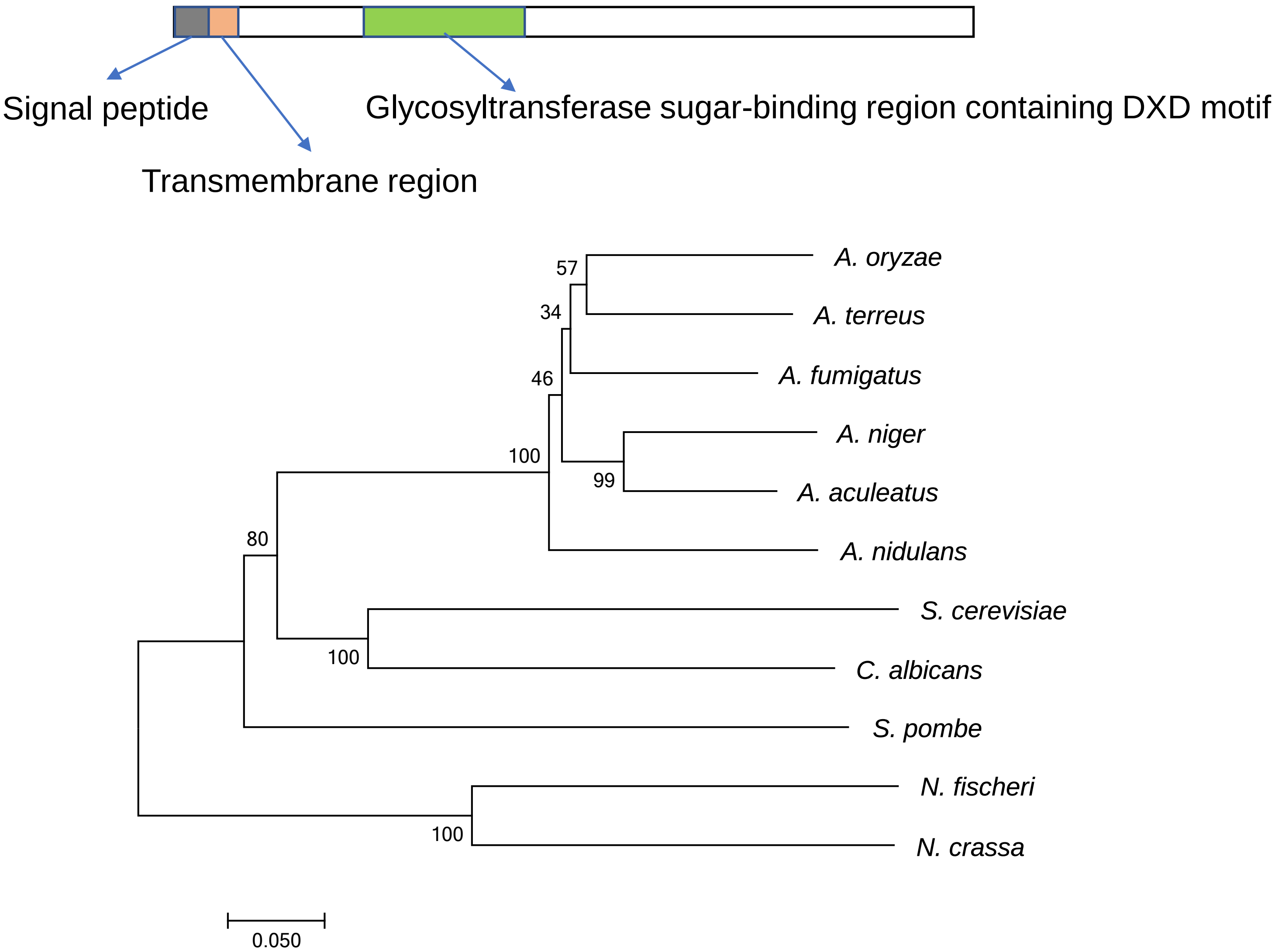

Fig. S4. Domain structure of the AoOch1 protein from *A. oryzae* and its phylogenetic tree

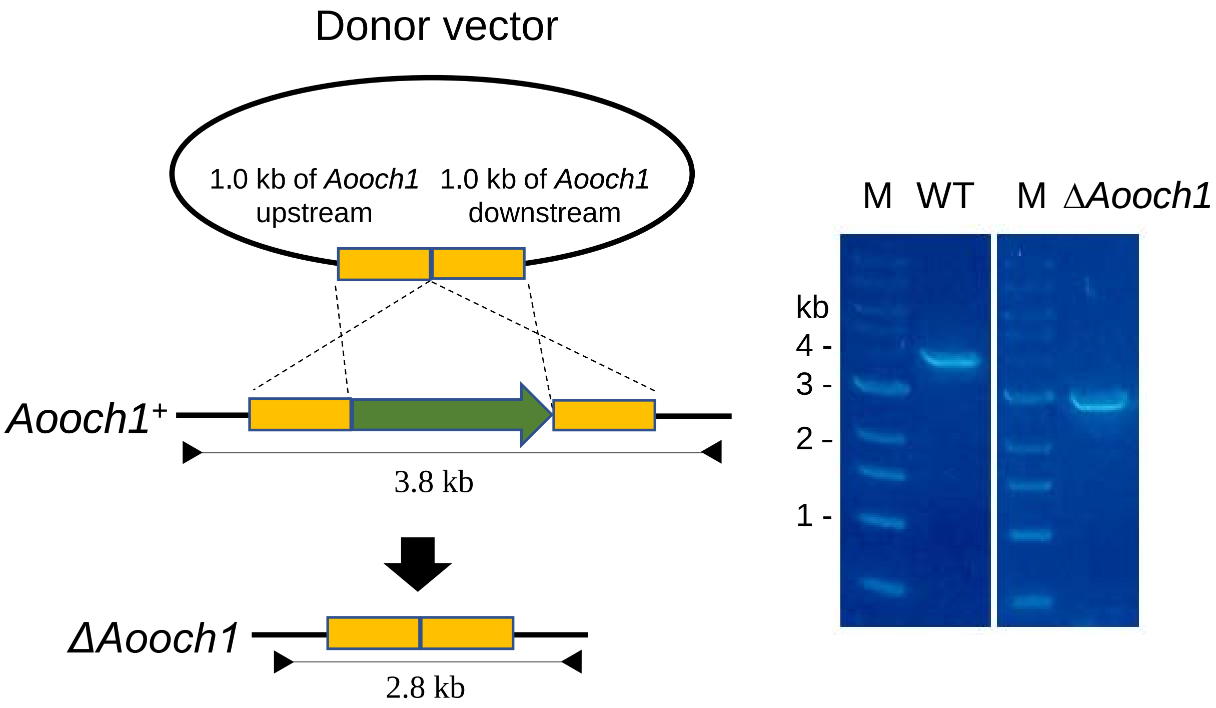

Fig. S5. Construction of CRISPR/Cas9 system for deleting the *Aooch1* gene and the colony PCR. WT and Δ*Aooch1* represent the wild-type NSlD-ΔP10 strain producing adalimumab and *Aooch1* gene deletion strain, respectively. M, DNA marker

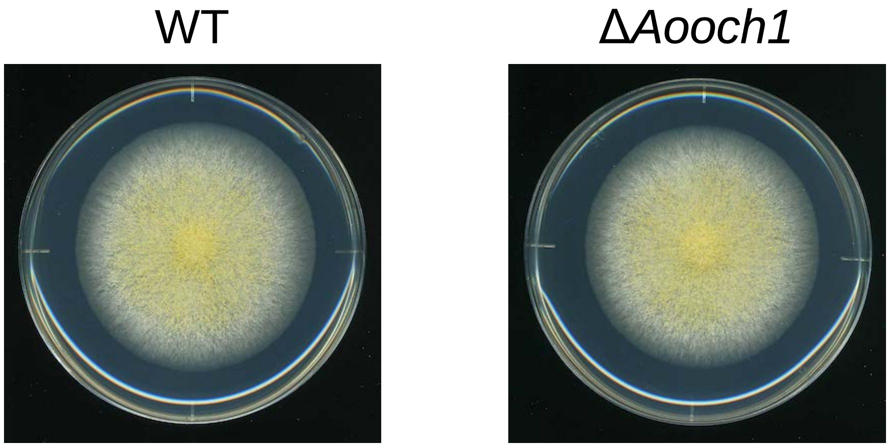

Fig. S6. Growth of Δ*Aooch1* strain. The conidial suspensions (2×10^6/5 μl) of *A. oryzae* WT and Δ*Aooch1* were inoculated onto the PD medium and incubated at 30°C for 4 days.

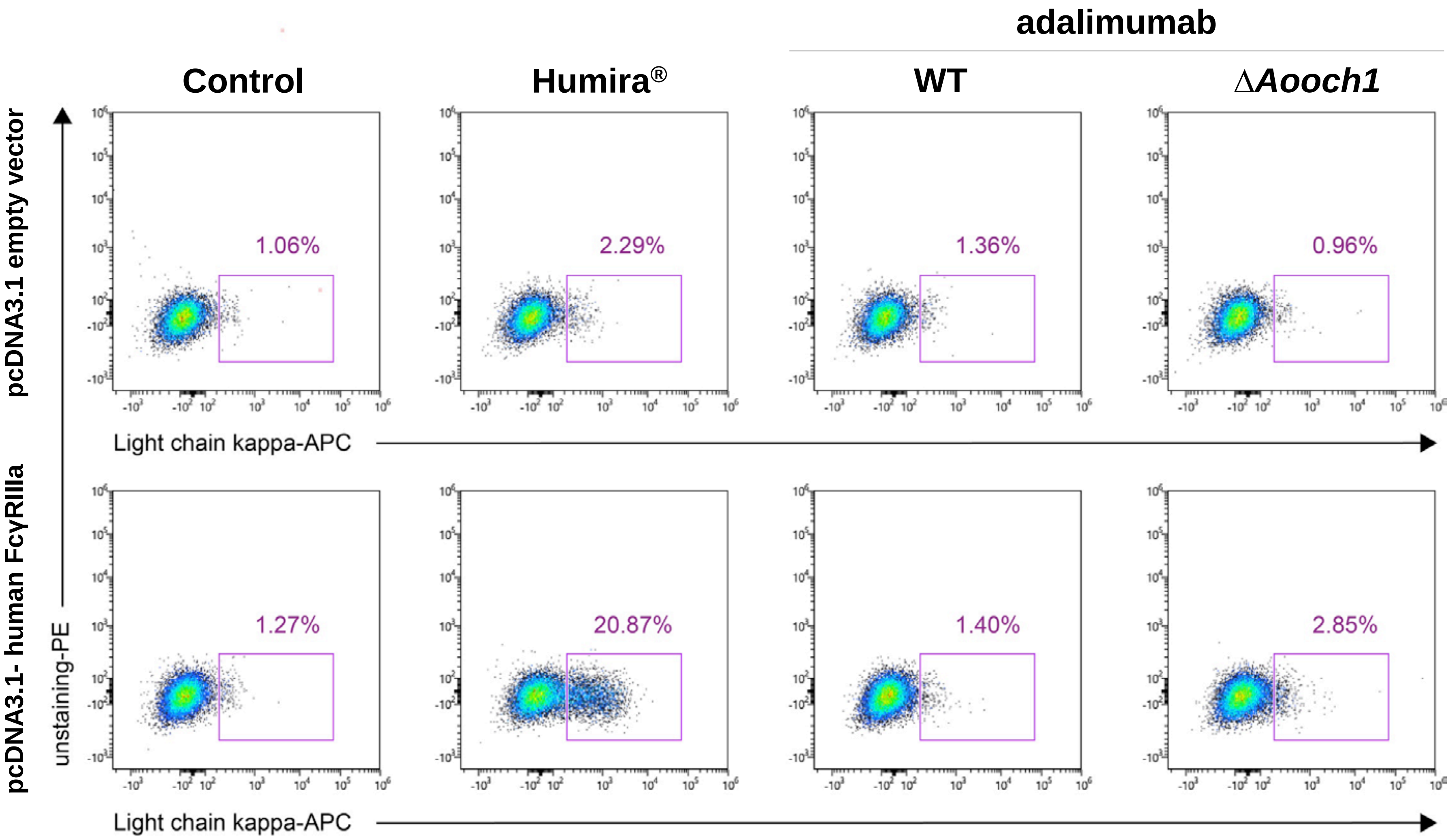

Fig. S7. The flow cytometry analysis of FcγRIIIa binding assay between Humira and adalimumab from *A. oryzae*.
